# Secondary Loss of Response to Dopamine Receptor Agonists in the Treatment of Macroprolactinoma

**DOI:** 10.7759/cureus.92046

**Published:** 2025-09-11

**Authors:** Alamin Alkundi, Rabiu Momoh

**Affiliations:** 1 Diabetes and Endocrinology, William Harvey Hospital, Kent, GBR; 2 Critical Care, Medway Maritime Hospital, Kent, GBR

**Keywords:** acquired resistance, doctors, dopamine receptor agonist, endocrinology, neurosurgery, patient education, pituitary gland, prolactin, prolactinoma, secondary loss of response to treatment

## Abstract

Secondary loss of response, sometimes termed "acquired resistance," refers to the phenomenon where a patient, initially responsive to DRA therapy, as evidenced by normalized prolactin levels and/or tumor reduction, experiences a recurrence of hyperprolactinemia and/or tumor regrowth despite continued therapy at previously effective doses. This differs from primary resistance, in which patients never respond to treatment.

We present an uncommonly described finding of a loss of response of macroprolactinoma treatment to cabergoline after an initial response in this case report. A literature review regarding primary and secondary resistance of prolactinomas to dopamine receptor agonist (DRA) therapy has been conducted. Patient education regarding the rare occurrence of secondary resistance of prolactinomas to DRA therapy can be better guided by this case report.

## Introduction

Prolactinomas arise from lactotrophic cells in the pituitary gland that secrete prolactin. They are the most common forms of pituitary tumors (accounting for up to 40%). They may exert functional effects because of hyperprolactinemia or a compressive/invasive effect in the macroprolactinoma type [1]. Dopamine naturally inhibits prolactin release, but in prolactinomas, this regulation is disrupted. Dopamine receptor agonists (DRAs) help restore balance by reducing prolactin levels (which alleviates symptoms like infertility, menstrual irregularities, and galactorrhea), shrinking tumor size, and serving as an initial alternative to surgical treatment. Knowledge about the medical treatment of prolactinomas with bromocriptine dates back to the 1970s, while cabergoline use dates back to the 1980s. Cabergoline is better favored in recent times in the treatment of prolactinomas because of its superior profile in normalizing prolactin level, reducing tumor size, and the convenience of a twice-a-week dosage [2].

Knowledge about primary resistance of prolactinomas to DRA therapy has received significant attention, but the occurrence of secondary resistance of prolactinomas to this class of drugs is infrequently described. Secondary resistance in prolactinomas refers to a loss of responsiveness to DRA therapy after an initial period of successful treatment. This is distinct from primary resistance, where the tumor never responded adequately to the medication ab initio [3]. We present a rare case of secondary resistance to prolactinoma therapy with cabergoline in a late septuagenarian. We have also reviewed available literature evidence regarding this rare concept for the benefit of the medical community.

## Case presentation

**Figure 1:**
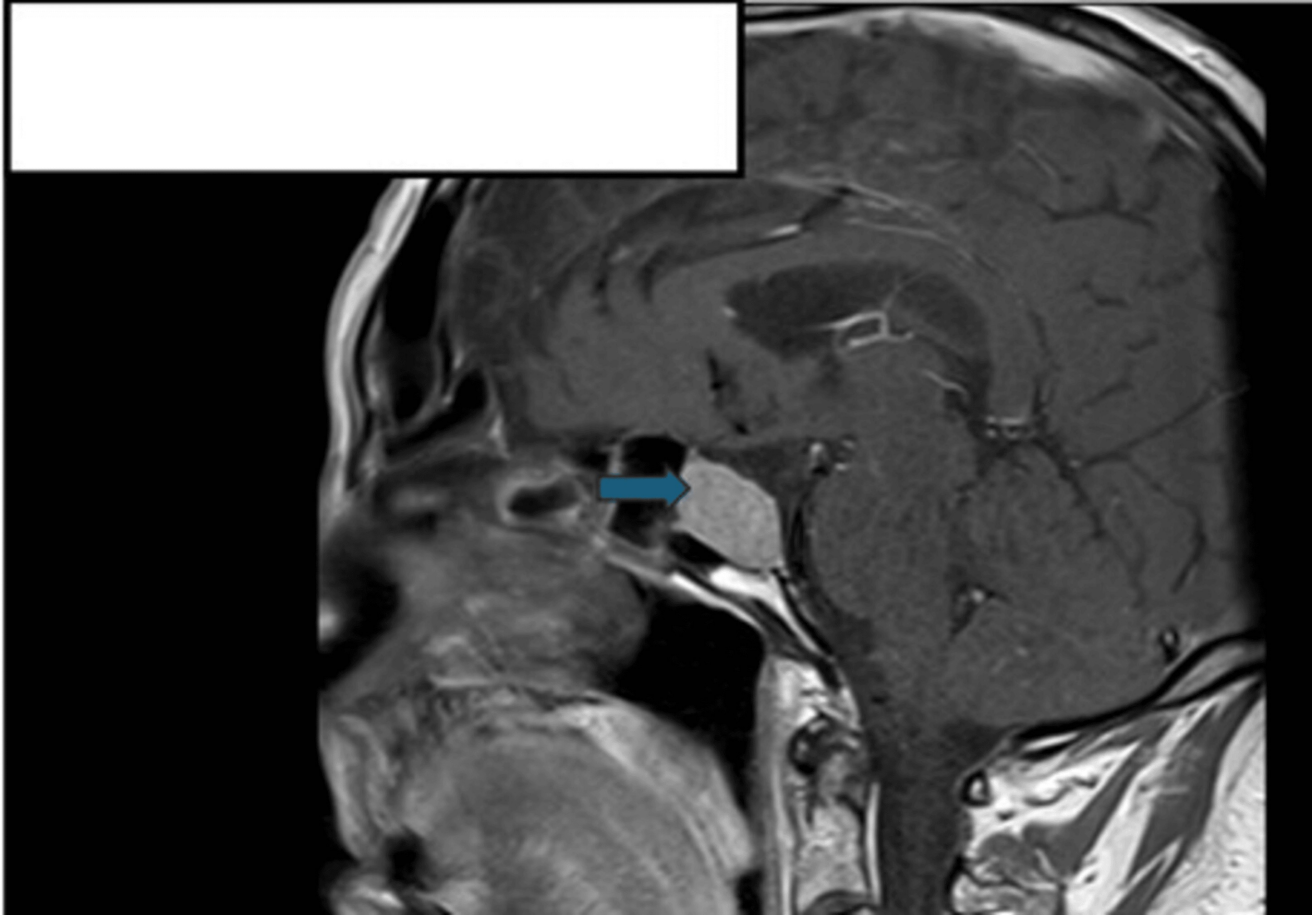
Sagittal section of a T1-weighted post-contrast MRI head scan performed eight years prior to this publication, revealing a possible macroadenoma (measuring 2.5 cm in maximum diameter) within the pituitary fossa. MRI: magnetic resonance imaging

**Figure 2:**
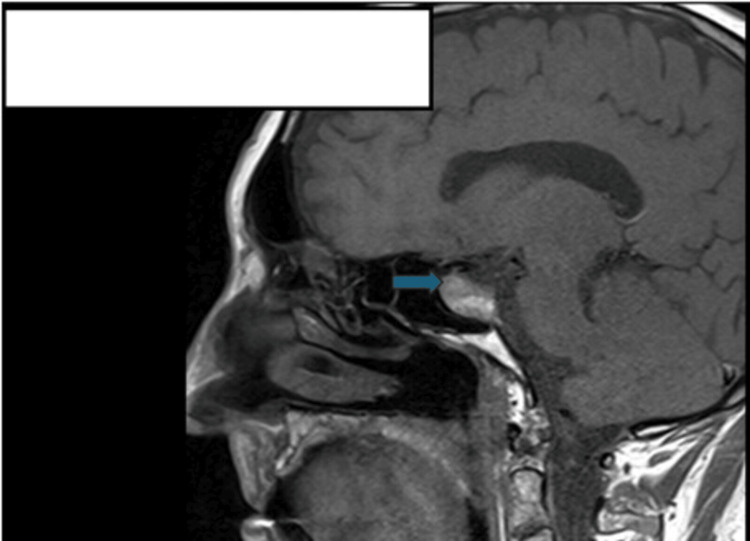
Sagittal section of a T1-weighted post-contrast MRI head scan performed three years prior to this publication, revealing a reduction in the size of the pituitary tumor (to 17×9 mm) with cabergoline treatment. MRI: magnetic resonance imaging

**Figure 3:**
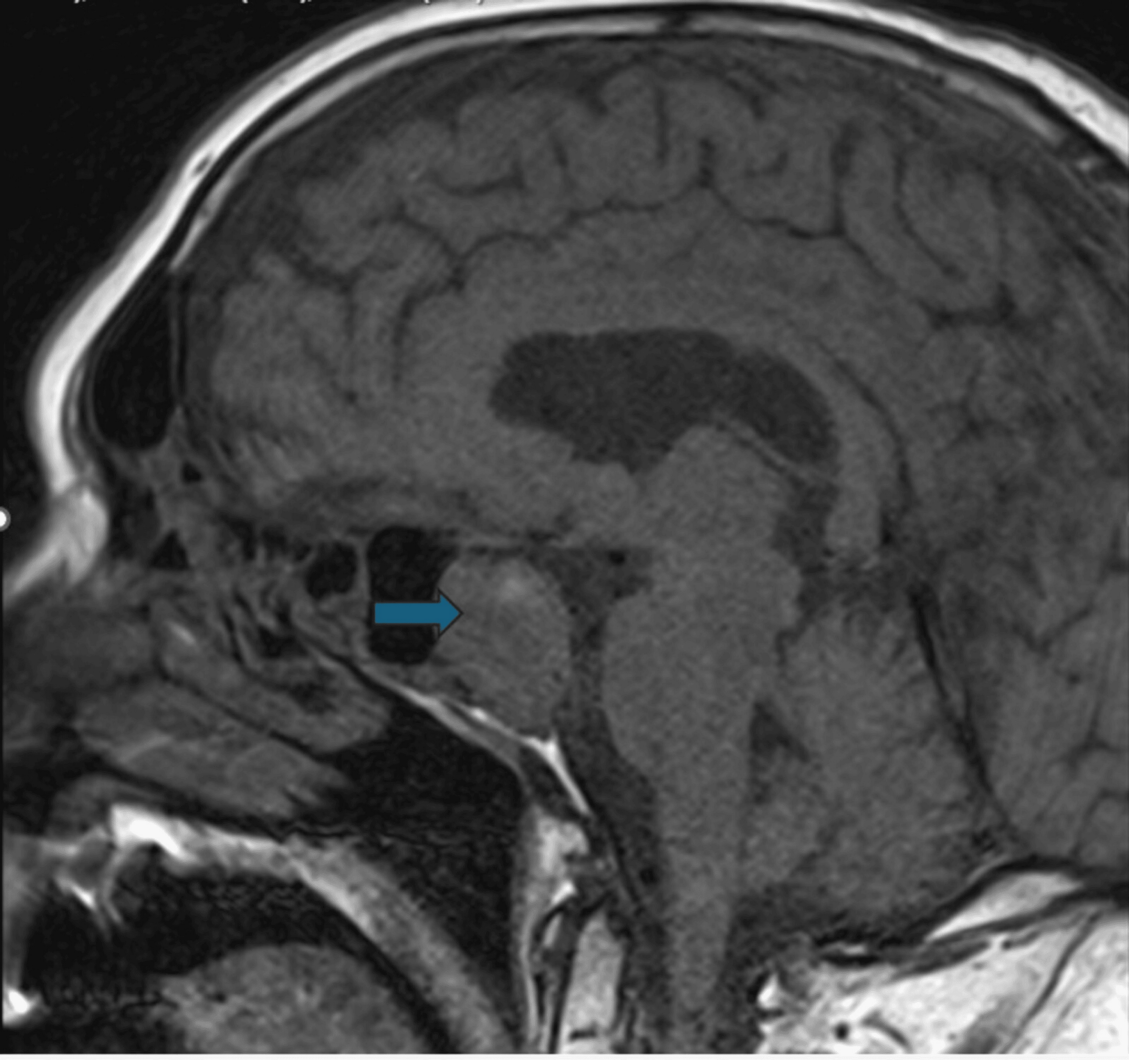
Sagittal section of a T1-weighted post-contrast MRI head scan performed one year prior to this publication, revealing an increase in the size of the pituitary tumor (to 20.5×26.8×22.3 mm) despite a previous reduction with cabergoline treatment.

We present the case of a 78-year-old male who was found to have a radiological finding of a 2.5 cm maximum diameter pituitary adenoma (Figure 1) while undergoing magnetic resonance imaging (MRI) for the assessment of non-specific Parkinsonian symptoms eight years prior. Around that time, he also noted recurrent headaches, fatigue, and loss of libido. Biochemistry studies performed then revealed a serum prolactin level of 22287 mU/L (ref: 86-324 mU/L) and an assessment of secondary hypothyroidism and hypogonadism (other hormonal results were not readily available at the time of this publication). He was initiated on oral cabergoline 250 µg twice weekly. The patient had a good response to cabergoline treatment, with a reduction in prolactin level to normal limits over an eight-month period and a reduction in the size of the prolactinoma over a five-year period of monitoring (Figure 2). However, over the following two years from the previous response to treatment, there was a notable increase in tumor size (Figure 3) and a progressive rise in serum prolactin to a new peak of 16002 mU/L (ref: 86-324 mU/L) despite treatment with an increased dose of cabergoline to 500 µg twice weekly (Table 1). An assessment of secondary loss of response of the prolactinoma to cabergoline treatment was made. He had periodic reviews in clinic and progressive up-titration of his cabergoline dose to 2 mg twice a week. See Table 2 for other serial hormone profile studies undertaken on the patient.

**Table 1:** Serial serum prolactin measurements in the patient, revealing secondary loss of response of the macroprolactinoma to cabergoline therapy.

Date of test	Result	Reference limit	Comment
10/05/2024	16002 mU/L	86-324 mU/L	Peak of secondary resistance to cabergoline therapy
06/04/2024	14762 mU/L	86-324 mU/L	
29/02/2024	10417 mU/L	86-324 mU/L	
17/1/2024	11403 mU/L	86-324 mU/L	
18/1/2023	1691 mU/L	86-324 mU/L	
10/3/2022	459 mU/L	86-324 mU/L	Gradual rise
15/9/2020	305 mU/L	86-324 mU/L	
22/7/2019	396 mU/L	86-324 mU/L	
17/10/2018	120 mU/L	86-324 mU/L	
4/6/2018	236 mU/L	86-324 mU/L	Normalization
29/5/2018	195 mU/L	86-324 mU/L	Initial normalization
24/11/2017	2797 mU/L	86-324 mU/L	
31/10/2017	22287 mU/L	86-324 mU/L	Onset of treatment with cabergoline

**Table 2:** Serial hormone profile studies performed on the patient. LH: luteinizing hormone, TSH: thyroid-stimulating hormone, ACTH: adrenocorticotropic hormone, IGF-1: insulin-like growth factor 1, FSH: follicle-stimulating hormone

Date	Test	Result	Reference	Comment
28/05/2025	Prolactin	9836 mU/L	86-324 mU/L	Markedly elevated
	Cortisol	224 nmol/L	140-690 nmol/L	Within normal limits
	Free T4	17.5 pmol/L	9-19 pmol/L	Within normal limits
	LH	0.8 IU/L	1.8 to 8.6 IU/L	Low
	TSH	0.8 mIU/L	0.4-5 mIU/L	Within normal limits
	ACTH	8 pmol/L	2 to 11 pmol/L	Within normal limits
	Growth hormone	0.11 ng/mL	<5 ng/mL	Within normal limits
	IGF-1	12.2 nmol/L	12-30 nmol/L	Low normal
10/5/2024	Prolactin	16002 mU/L	86-324 mU/L	Markedly elevated
	9 am cortisol	421 nmol/L	140-690 nmol/L	Within normal limits
6/04/2024	Prolactin	14762 mU/L	86-324 mU/L	Markedly elevated
	TSH	0.8 mIU/L	0.4-5 mIU/L	Within normal limit
	Free T4	11 pmol/L	9-19 pmol/L	Within normal limits
17/1/2024	Testosterone	24.2 nmol/L	8.7 to 29 nmol/L	Within normal limits
	Prolactin	459 mU/L	86-324 mU/L	Mildly elevated
30/10/2017	TSH	1.1 mIU/L	0.4-5 mIU/L	Within normal limits
	Free T4	7 pmol/L	9-19 pmol/L	Mildly low
	Estradiol	<50 pmol/L	Male reference: <160 pmol/L	Within normal limit
	FSH	2 IU/L	1-12 IU/L	Within normal limits
	Prolactin	22287 mU/L	0-700 mU/L	Markedly elevated
	9 am cortisol	400 nmol/L	140-690 nmol/L	Within normal limits
	Testosterone	0.9 nmol/L	8.7 to 29 nmol/L	Low

On review at a recent clinic visit, his other past medical history included benign paroxysmal positional vertigo, secondary hypogonadism, benign prostatic hypertrophy, gout, and syndrome of inappropriate anti-diuretic hormone (SIADH). His regular medications, apart from cabergoline treatment, included oral testosterone, finasteride, tamsulosin, lansoprazole, allopurinol, fluoxetine, and co-codamol. He was a retired builder. On physical examination, he was not very steady on his feet and reported recurrent falls. He had no visual disturbances or galacturia. His blood pressure was 115/72, pulse 82, and weight 82.5 kg. Heart sounds were faint but normal; his chest was clear, and his abdomen was soft and non-tender with no obvious palpable masses. His color vision assessment was 15/17 in both eyes. Optical coherence tomography (OCT) performed revealed no optic disc pallor or swelling. The patient’s visual fields appeared normal during the confrontation test. Intraocular pressure measured 8 mmHg in the right eye and 9 mmHg in the left eye (ref: 10-16 mmHg). He had a 6/18 visual acuity score in both eyes with glasses and the pinhole test. No other cranial nerve deficits were found on examination.

His latest MRI-pituitary scan showed a pituitary macroadenoma measuring 20.5×26.8×22.3 mm (Figure 3), with a corresponding serum prolactin level of 16002 mU/L (markedly elevated, suggesting marked secondary resistance to cabergoline therapy), compared with a previous measurement of 14.3×20.1×22 mm measured five months prior (with a corresponding serum prolactin value of 11403 mU/L (elevated)). The optic chiasma was deviated upward and showed compression. There was a small area of T1 hyperintensity in the upper part of the macroadenoma, suggesting a subacute bleed. The MRI-pituitary scan performed three years prior showed a reduction to 17×9 mm in size (see Figure 2), with a corresponding serum prolactin value of 459 mU/L (moderately raised, on treatment with cabergoline), compared with an assessment of 21 × 10 mm done six years prior to this case submission (with a corresponding serum prolactin level of 396 mU/L).

The decision was made to undertake surgical treatment of his macroprolactinoma in the face of secondary resistance to cabergoline therapy, even at escalated doses. He underwent trans-sphenoidal hypophysectomy and abdominal fat harvesting, which was transplanted into the pituitary fossa to prevent cerebrospinal fluid leak. Histology of the excisional biopsy revealed a lactotroph PitNet/adenoma with an increased proliferative index; Ki-67 of 5%. He had no major complications post-surgery and was subsequently discharged home on oral hydrocortisone 10 mg twice a day. Oral cabergoline was discontinued. He was planned for a pituitary hormone level check and an insulin tolerance test (IST) at six weeks postoperatively and an MRI pituitary scan at three months post-surgery.

## Discussion

Prolactinomas are the most frequently occurring hormone-secreting pituitary tumors, marked by an overproduction of prolactin. The primary treatment approach involves DRAs like bromocriptine and cabergoline. These medications typically yield excellent results, including normalized prolactin levels and notable tumor reduction. Nonetheless, some patients later lose responsiveness to DRA therapy after initially benefiting from it, posing a considerable clinical dilemma [3]. We present the rare finding of a secondary loss of response of macroprolactinoma in our patient to cabergoline therapy.

The exact mechanisms driving secondary resistance are not fully elucidated, but several hypotheses and contributing factors have emerged.

Molitch (2003), in a published article, explained the concept of dopamine resistance of prolactinoma as a failure to achieve a drop to normal prolactin level or a reduction of the size of the prolactinoma by at least 50% with DRA treatment. He noted that failure to achieve a normal prolactin level is more likely with bromocriptine than pergolide, and even less so with cabergoline (the agent in focus in this case report). He also noted that failure to achieve a reduction in prolactinoma size was more likely with bromocriptine than with pergolide or cabergoline [4].

The notable difference in our case report was the initial achievement of a drop in prolactin level to near-normal levels and a reduction in the size of the prolactinoma with cabergoline treatment, followed by a loss of response to this medication, accompanied by a further rise in prolactin level and a notable increase in tumor size on a subsequent MRI study. Important confounding factors, such as the patient’s adherence to medication, were considered but deemed unlikely, as the patient and his partner attested to judicious use of prescribed medications. Issues regarding assay variation or laboratory reliability were deemed unlikely, as samples were analyzed consistently in the same laboratory. Molitch further suggested that a reduction in the number of D2 dopamine receptors (though having good affinity) is responsible for the primary dopamine resistance of prolactinomas in the first instance [4].

Mallea-Gil et al. (2009) published the mortal case of a septuagenarian who developed secondary resistance of a macroprolactinoma to cabergoline treatment. Despite the patient undergoing transcranial surgery for tumor excision, they noted tumor recurrence and persistently elevated prolactin levels. They also reported a late rise in growth hormone and insulin-like growth factor 1 (IGF-1) in the tumor’s late stages. They noted high vascularization of the excised adenoma on immunochemistry, revealing strong expression of vascular endothelial growth factor (VEGF), fibroblast growth factor (FGF-2), and CD31 [5].

Shimon, in his 2023 publication, acknowledged that the development of secondary resistance by prolactinomas is rare. He further stated that rare malignant transformation of prolactinomas could be responsible for secondary resistance. He noted that prolactinomas fall behind corticotrophic adenomas in terms of malignant transformation and that distant metastasis of malignant pituitary tumors is rare [6]. Histologic analysis of the excised tumor in our patient revealed a lactotroph pituitary adenoma with a Ki-67 index of 5%, suggesting increased proliferative capacity but not definitively indicative of malignancy. While a Ki-67 index of 5% is higher than the typical range for benign pituitary adenomas, it does not automatically classify the tumor as aggressive or malignant. Our patient is scheduled for further follow-up assessments.

Delgrange et al. (1998) reported a secondary loss of response to bromocriptine in the treatment of a macroprolactinoma that was initially responsive. The patient’s treatment was switched to quinagolide and later to cabergoline. They noted no further tumor growth in contrast to this index case report. They suggested that malignant transformation was unlikely and that the loss of dopamine receptors on the surface of prolactinoma cells may underlie secondary resistance to bromocriptine [7].

Shimon, in the same publication, reviewed features that could account for refractoriness of prolactinomas to DRA therapy. He noted that younger males, tumors with higher proliferative rates, lower expressions of D2 receptors, low estrogen receptor-alpha (ER-alpha), reduced filamin-A expression, down-regulation of the TGFβ/Smad signaling cascade, somatic hotspot SF3B1 and POU6F2 mutations, short-to-long D2 dopamine receptor isoform ratios, and the activity of Gi/Go proteins coupled to adenylate cyclase could predispose prolactinomas to being refractory to DRAs [6].

Shimon also went on to suggest the use of a multi-modal approach in the management of prolactinomas that are refractory to DRA therapy, and this should include considering a switch to higher doses of cabergoline, considering pituitary surgery, and, if possible, undertaking radiotherapy to manage residual prolactinoma tumor cells. He further highlighted other experimental therapies that are being considered for the management of refractory prolactinomas to include the use of pasireotide, immune checkpoint inhibitors (such as nivolumab and ipilimumab), tyrosine-kinase inhibitors, or aromatase inhibitors [6].

Molitch suggested considering a switch to a different DRA when resistance occurs, increasing the dose of the ongoing DRA, or considering surgery. He also noted that DRA resistance in prolactinomas may be acceptable if no further tumor growth occurs [4]. In our case, however, a notable increase in prolactinoma size was observed despite the initial reduction with cabergoline. A trial of cabergoline dose escalation was attempted, but it did not reverse secondary tumor growth or further suppress serum prolactin levels, prompting consideration of surgical intervention for our patient.

Murakami et al. (2011) described the fatal case of a sextuagenarian female previously diagnosed with an atypical prolactinoma who had undergone various treatments over seven years (dopamine agonists, five surgeries, conventional radiotherapy, and radiosurgery). Despite these interventions, she experienced tumor regrowth and clinical deterioration. After ten cycles of temozolomide (TMZ), her condition improved, and the tumor shrank. Six months after stopping TMZ, the tumor progressed to pituitary carcinoma with regrowth and notable ventricular extension. Further TMZ treatment was ineffective. A sixth surgery and subsequent salvage chemotherapy did not improve her condition. The atypical prolactinoma and pituitary carcinoma exhibited a p53 mutation. The mismatch repair protein MSH6 was present in the atypical adenoma but absent in the TMZ-resistant pituitary carcinoma [8].

Secondary resistance of prolactinomas to DRA is relatively uncommon, with estimates ranging from 2% and 10% of treated patients, depending on the cohort and treatment duration. Prognosis varies. Patients with microprolactinomas or non-invasive tumors typically retain more treatment options and better outcomes. Aggressive or invasive macroprolactinomas resistant to DRAs may require multimodal therapy and long-term follow-up [9].

Future research in this field should aim to better understand the molecular basis of DRA resistance, develop novel drugs targeting alternative pathways involved in tumor growth, identify biomarkers to predict and monitor resistance, and personalize therapy based on tumor genetics and patient phenotype [10].

## Conclusions

This case report highlights the importance of awareness regarding secondary resistance of prolactinomas to DRA therapy, its management challenges, and the need for a multimodal approach in prolactinoma care. Multimodal treatment strategies may include consideration of higher tolerable doses of cabergoline, trans-sphenoidal surgery, and, where appropriate, radiotherapy for managing this rare scenario of secondary DRA resistance. Physicians should be mindful of this uncommon occurrence, and patient education can help facilitate understanding of this rare complication.
